# Olaparib Monotherapy for *BRIP1*-Mutated High-Grade Serous Endometrial Cancer

**DOI:** 10.1200/PO.19.00368

**Published:** 2020-04-09

**Authors:** Kohei Nakamura, Eriko Aimono, Shigeki Tanishima, Mitsuho Imai, Akiko Kawano Nagatsuma, Hideyuki Hayashi, Yuki Yoshimura, Kentaro Nakayama, Satoru Kyo, Hiroshi Nishihara

**Affiliations:** ^1^ Genomics Unit, Keio Cancer Center, Keio University School of Medicine, Tokyo, Japan; ^2^ Department of Obstetrics and Gynecology, Kumagaya General Hospital, Saitama, Japan; ^3^ Department of Biomedical Informatics, Kansai Division, Mitsubishi Space Software, Tokyo, Japan; ^4^ Department of Obstetrics and Gynecology, Shimane University School of Medicine, Izumo, Japan

## INTRODUCTION

The prognosis for women with high-grade endometrial cancers is poor, with little improvement in the last two decades.^1^ The mainstay of treatment is surgery (hysterectomy) with or without lymphadenectomy. Although adjuvant radiotherapy is considered standard for high-risk endometrial cancers, the added value of chemotherapy has been the subject of recent trials. However, biomarkers predicting chemotherapeutic benefits have not been defined. *BRCA1* and *BRCA2* encode proteins that repair breaks in double-stranded DNA via the homologous recombination repair (HRR) pathway, and mutations in these genes cause HRR deficiency. Recently, other mutations were reported in ovarian cancers, such as those in Fanconi anemia genes (eg, *PALB2* [*FANCN*], *BRIP1* [*FANCJ*], and *RAD51C*) and other genes involved in HRR (eg, *ATM*, *BARD1*, *NBN*, *CDK12*, *TP53*, and *CHEK2*).^2,3^

Poly (ADP-ribose) polymerase (PARP1) is a DNA repair enzyme involved in base excision and single-stranded break repair pathways.^4^ PARP1 inhibition in HRR-deficient cells blocks single-stranded DNA break repair pathways, leaving only double-stranded DNA break repair pathways functional. This results in synthetic lethality from the loss of homologous recombination and base excision repair, thus activating highly error-prone DNA repair pathways, eventually causing cell death.^5,6^ PARP inhibitors also trap PARP-DNA complexes at the replication fork, increase toxic nonhomologous end joining in PARP1-deficient cells, and block PARP1/Polθ-mediated alternative end joining.^7^

The US Food and Drug Administration (FDA) has approved three PARP inhibitors (olaparib, rucaparib, and niraparib) as monotherapies for breast and ovarian cancers. However, no such drugs are recommended or approved for endometrial cancers. This study describes a case in which olaparib was used to treat a patient with advanced serous endometrial carcinoma with a *BRIP1* mutation.

## RESULTS

**FIG 1.**
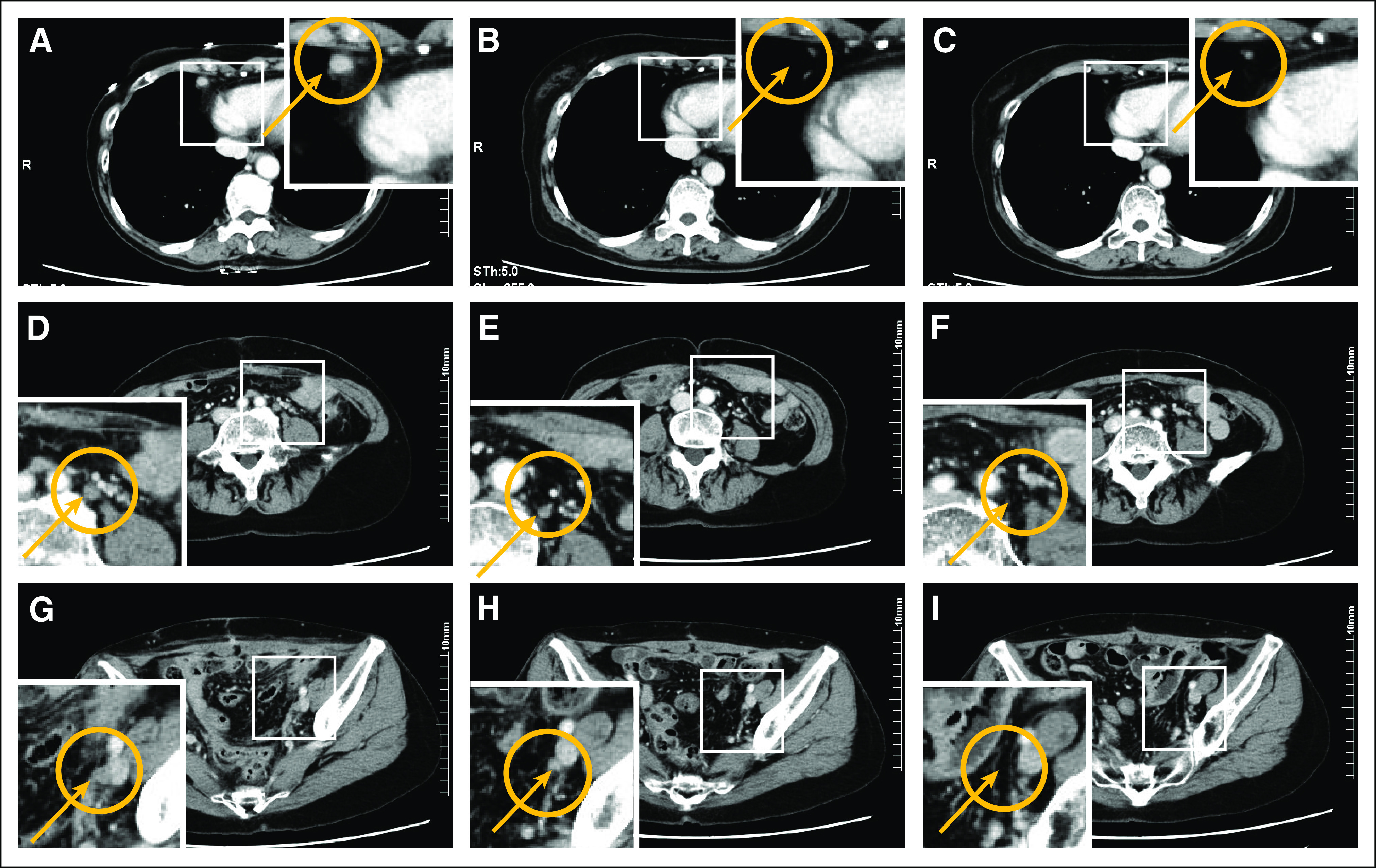
Shrinkage of the patient’s (A-C) cardiophrenic, (D-F) para-aortic, and (G-I) left lateral iliac lymph nodes after 3 months of olaparib treatment. Computed tomographic imaging of the abdomen and pelvis (A, D, G) before treatment, (B, E, H) after second-line chemotherapy, and (C, F, I) after olaparib treatment.

A 70-year-old woman with a family history of colorectal and endometrial cancers experienced abnormal vaginal bleeding. Endometrial biopsy revealed high-grade serous carcinoma of the endometrium. Pelvic magnetic resonance imaging revealed a thickened endometrium, measuring 4.2 × 4.0 cm. Computed tomography (CT) revealed multiple swollen cardiophrenic, para-aortic, and left lateral iliac lymph nodes and omental cake (Fig 1). The patient underwent total abdominal hysterectomy, bilateral salpingo-oophorectomy, and omentectomy. Resected tissues were examined by a pathologist, who diagnosed the mass as stage IVB (T1bNxM1) high-grade serous endometrial carcinoma. The tumor measured 4.9 × 4.6 cm and had invaded the myometrium to a depth of 95%; there was also evidence of lymphovascular space invasion. Immunohistochemical analysis revealed normal mismatch repair protein expression, suggesting that this was not a familial endometrial cancer (Lynch syndrome). P53 immunostaining was also positive, suggesting a *TP53* mutation. After surgery, the patient received six cycles of carboplatin and paclitaxel.

After first-line chemotherapy, CT indicated stable disease based on RECIST 1.0 criteria, with only improvements in the cardiophrenic swollen lymph node (Fig 1). The patient then started doxorubicin and cisplatin for second-line chemotherapy. After six cycles, CT revealed essentially stable disease without improvement in the para-aortic or left lateral iliac swollen lymph nodes (Fig 1).

**FIG 2.**
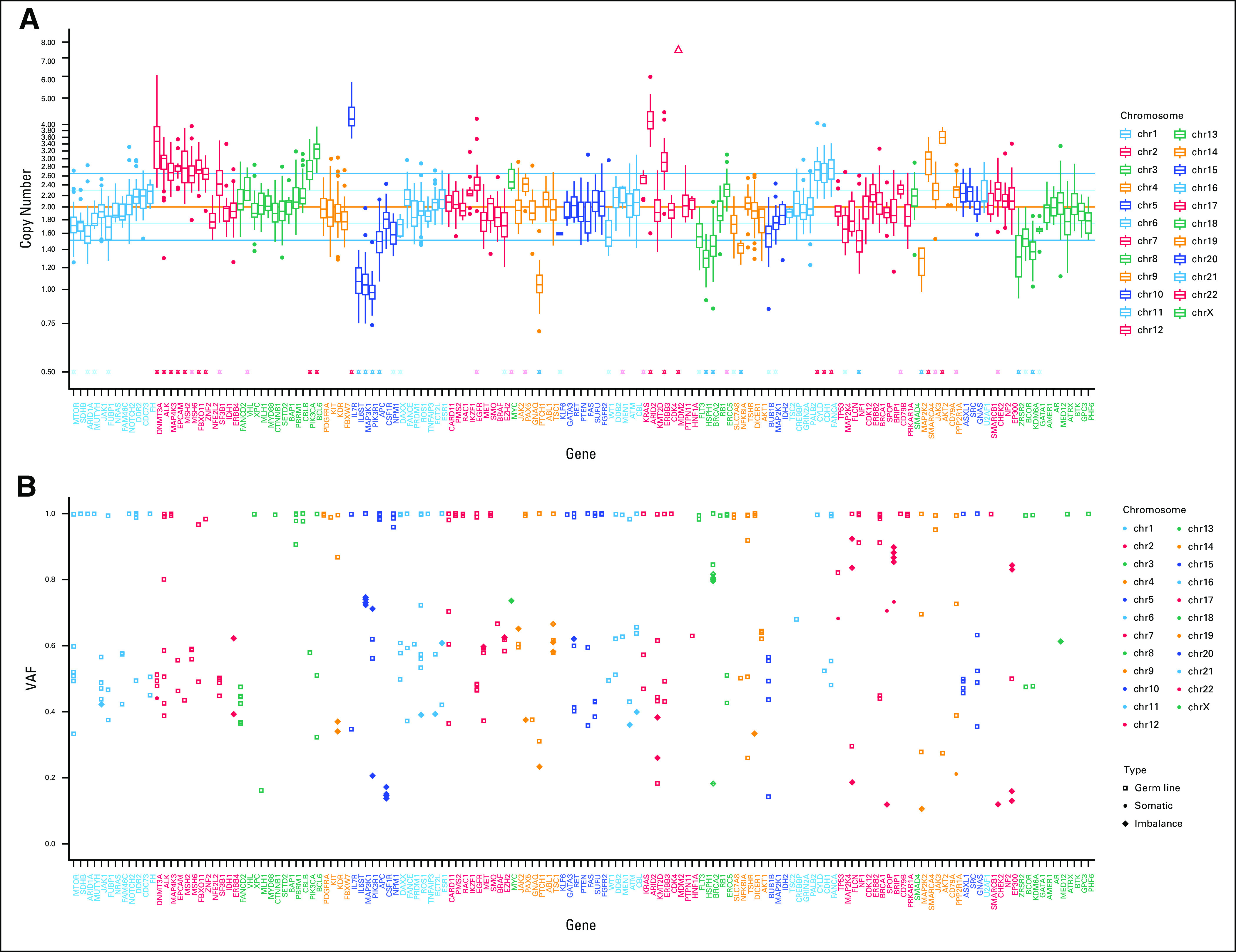
(A) Copy-number alteration and (B) variant allele frequency (VAF) indicating homologous recombination deficiency pattern. The horizontal axis corresponds to each examined gene, and the vertical axis corresponds to the (A) copy number or (B) VAF.

**FIG A1.**
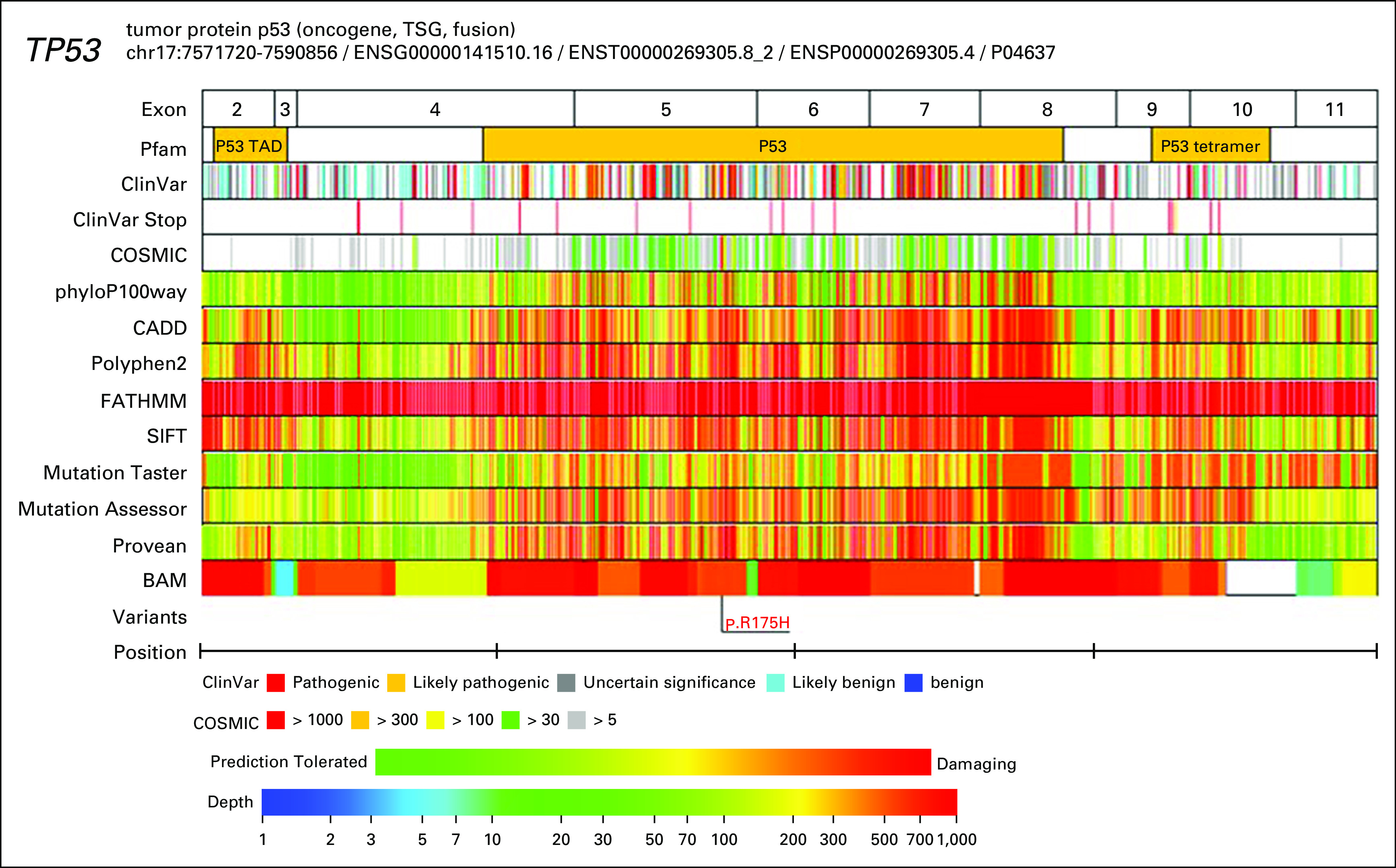
Details of the *TP53* point mutation.

**FIG A2.**
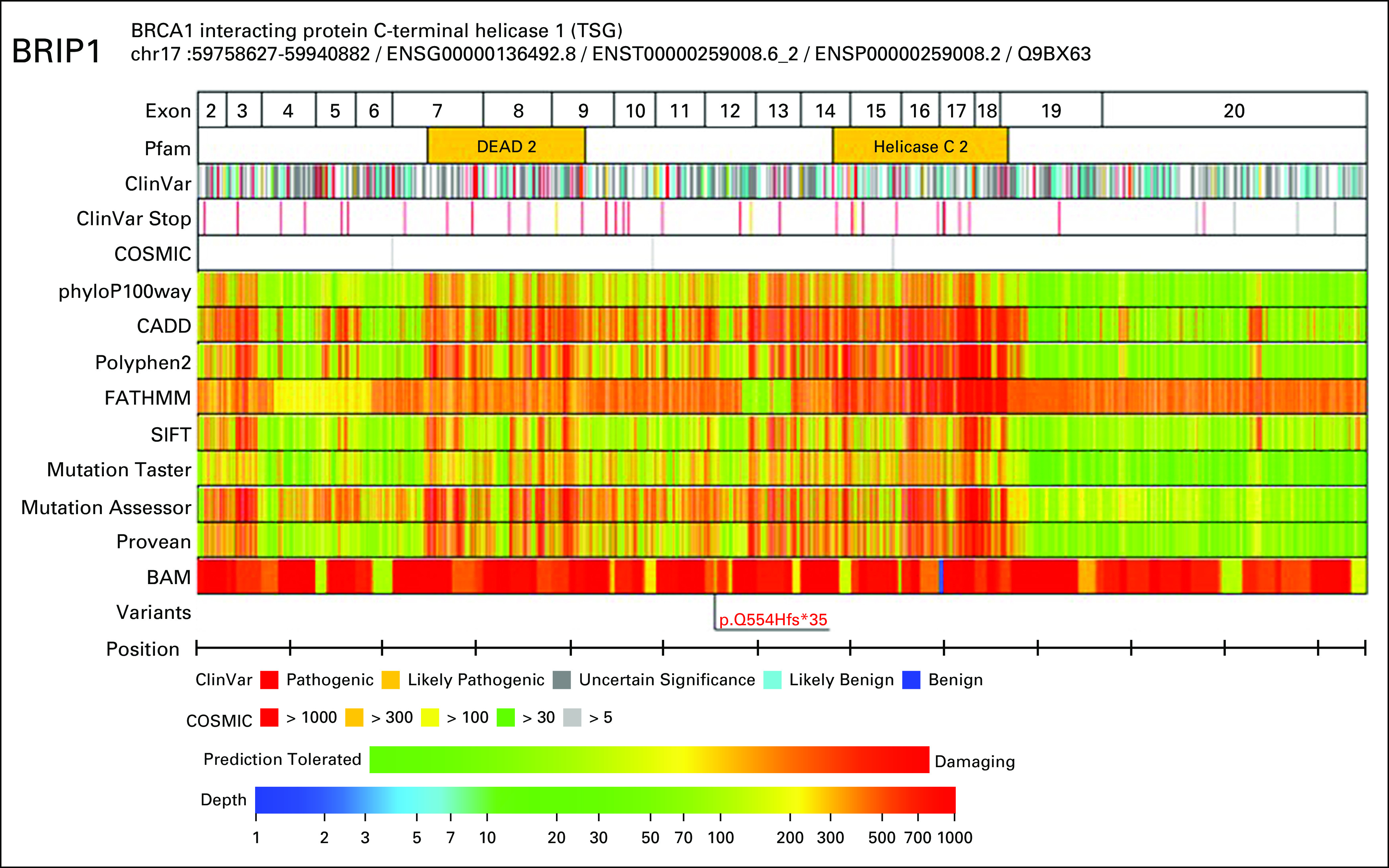
Details of the frameshift *BRIP1* alteration.

Targeted next-generation sequencing of the patient’s blood and resected specimen was performed using an in-house assay during treatment. A *TP53* somatic point mutation (p.R175H) and somatic frameshift *BRIP1* (p.Q554Hfs*35) alterations were detected as pathogenic variants in the tumor; detailed information is provided in Appendix Figures A1 and A2. All detected variants, including variants of unknown significance are presented in Appendix Table A1. Loss of genetic heterozygosity (LOH) without mutation was observed in *BRCA2*, *MAP3K1*, *PIK3R1*, *PTCH1*, and *KDM6A*. No gene amplification was detected. Tumor mutation burden calculated from our pipeline was 1.3 single-nucleotide variants per megabase in the samples. Copy-number variation box and variant allele frequency plots (Fig 2) indicated a high LOH frequency and scattered allelic imbalance, which are often detected in homologous recombination–deficient tumors. Secondary germ line examination found no American College of Medical Genetics and Genomics–recommended genes for testing.

Given her somatic *BRIP1* mutation and findings indicating LOH high status, the potential efficacy of the PARP inhibitor olaparib was discussed with the patient. The 300-mg oral twice-daily dose recommended by the FDA was initiated off label, and her progress was observed closely. A CT scan 3 months after olaparib initiation showed the disappearance of all residual swollen lymph nodes in the pelvis (Fig 1). No adverse events were observed, including anemia, neutropenia, thrombocytopenia, nausea, and fatigue. The patient continued to receive olaparib and underwent close clinical follow-up every 2 months. She completed > 9 months of olaparib and showed complete response on her most recent CT.

## DISCUSSION

Endometrial cancers are categorized into two main histologic types.^8^ Type I endometrial tumors show endometrioid histology and typically express estrogen (ER) and progesterone (PR) receptors. In contrast, type II endometrial tumors do not exhibit endometrioid histology (predominantly serous histology) and are associated with poorer prognoses, with a 5-year overall survival rate of 55%.^1^ Type II tumors neither express ER/PR nor respond to endocrine therapy. *TP53* mutations are observed in > 90% of type II tumors,^9^ with mutations occurring commonly in *PIK3CA*, *ERBB2*, *FBXW7*, *PPP2R1A*, and *CCNE1*. Meanwhile, type I tumors exhibit significantly more PI3K pathway alterations (primarily *PTEN* and *PIK3CA* mutations).^9,10^ In our case, next-generation target panel sequencing results reflected the molecular events observed in serous endometrial cancers, such as *TP53* mutations.

Whereas BRIP1 is implicated in double-stranded DNA break repair via HRR pathways, the frequency of *BRIP1* mutations in type II endometrial tumors remains unclear. In a previous study by Heeke et al,^11^ the prevalence of homologous recombination–related gene mutations across multiple cancer types was investigated in 52,426 malignant tumors. They reported that the overall frequency of HRR alterations detected was 17.4%, and the *BRIP1* mutation was detected in only 0.2% of all tumors and 0.14% of endometrial cancers. Regarding other homologous recombination–related genes, *ARID1A* was detected in 7.2%, *BRCA1* in 2.8%, *BRCA2* in 3.0%, *ATM* in 1.3%, *PALB2* in 0.6%, and *FANCC* in 0.1% of all tumors. As in ovarian cancer, HRR alterations are also observed, although at a lower frequency, in other human malignancies, including melanoma, breast, pancreatic, and prostate cancers. This has promoted clinical studies of PARP inhibitors in contexts other than ovarian cancer. Moreover, their potential therapeutic applications might be extended from germ line *BRCA* mutations to target a more diverse group of sporadic tumors, such as those with epigenetic disruption of *BRCA1*/*2* function or genetically or epigenetically acquired aberrations in other important HRR pathway constituents.^12^ There is clinical evidence to support this theory, with olaparib approved by the FDA as an alternative for patients with germ line or somatic *BRCA1*/*2* mutations and as maintenance therapy after platinum-based chemotherapy in platinum-sensitive recurrent epithelial ovarian carcinoma, regardless of *BRCA* mutation status.^13^ HRR alterations are associated with *BRCA1*/*2* mutations in high-grade serous endometrial cancers; however, PARP inhibitors await FDA approval for this indication, and there are currently no trials of PARP inhibitors in patients with endometrial cancers with *BRCA* or other HRR-associated genealterations.

Accumulating evidence indicates that patients with *BRIP1* mutations have HRR deficiency and are consequently hypersensitive to PARP inhibition.^14^ BRIP1 is a BRCA1-interacting protein (the BRIP1-BRCA1 interaction is important for HRR) and associates with BRCA1 as cells progress through the S phase of the cell cycle.^15,16^ BRIP1 is a DNA helicase that interacts with the COOH-terminal BRCT repeat of BRCA1. BRIP1 is associated with the GM1/2 checkpoint, as well as the activation of CHK1, regulation of entry into the S phase, and maintenance of genomic stability.^16^ Thus, if the complex involving BRCA1 and BRIP1 is involved in tumor suppression, mutations in the genes that encode these proteins should be associated with altered cancer risk. Our data suggest that the *BRIP1* mutations could indicate the response to PARP inhibition, thus supporting our hypothesis.

Furthermore, variant allele frequency plots (Fig 2) indicated the potential of a high LOH frequency and scattered allelic imbalance in this case. Recently, three independent DNA-based measures of genomic instability reflecting underlying tumor homologous recombination DNA repair deficiency were developed based on LOH, telomeric allelic imbalance, and large-scale state transitions.^17-19^ However, we could not determine the homologous recombination deficiency score, because the relevant test is not covered by insurance in Japan, and therefore, we could not prove this. However, we can hypothesize that the *BRIP1* mutation results in HRR deficiency and subsequently causes a high LOH frequency and scattered allelic imbalance, potentially resulting in good response to PARP inhibitors.

In conclusion, we describe the case of a 70-year-old woman exhibiting a durable clinical radiographic response to olaparib. The patient had serous endometrial cancer harboring a somatic *BRIP1* mutation and had exhausted standard treatment alternatives. We demonstrate that PARP inhibitors have potential in treating endometrial cancers with *BRIP1* mutations. Targeted sequencing could also be used for the clinical management of patients with endometrial cancers.

## Figures and Tables

**TABLE A1.**
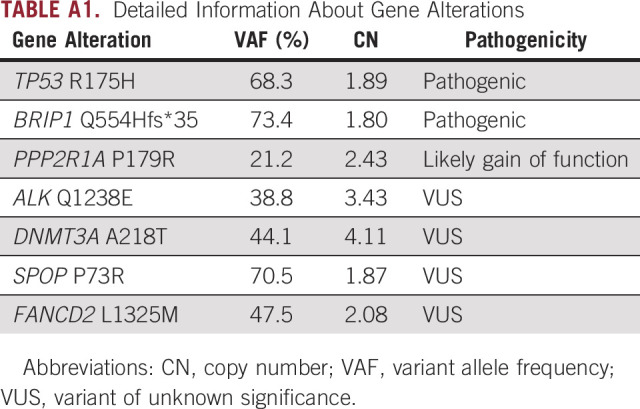
Detailed Information About Gene Alterations

**TABLE A2.**
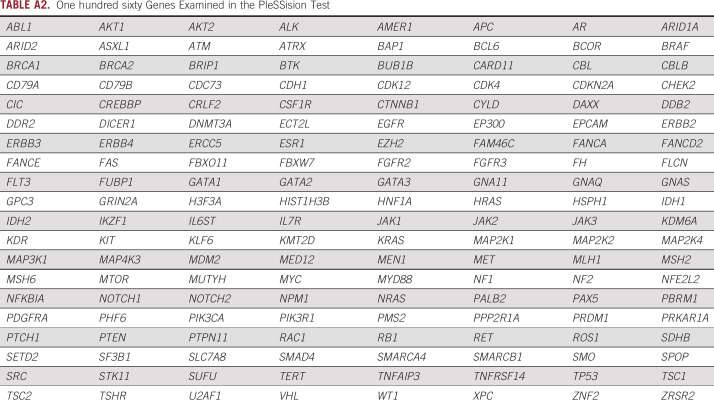
One hundred sixty Genes Examined in the PleSSision Test

## Appendix

We investigated the occurrence of mutations in 160 cancer-related genes in the endometrial tumor. Sections (10 μm) were dissected to provide > 20% tumor cells in the specimens and minimize the presence of necrosis. Genomic testing was performed on a PleSSision internal clinical sequencing apparatus (Keio University, Tokyo, Japan), which is used for all genome sequencing–related analyses in our hospital (Keio University Hospital). This apparatus was used to extract genomic DNA from tumor samples and peripheral blood mononuclear cells extracted from patients with cancer, after the provision of consent to undergo comprehensive genomic testing. This study was conducted in accordance with the Declaration of Helsinki and Title 45, US Code of Federal Regulations, Part 46, Protection of Human Subjects, effective December 13, 2001.

DNA quality was checked by calculating the DNA integrity number (DIN) using an Agilent 2000 TapeStation (Agilent Technologies, Waldbronn, Germany) before conducting targeted amplicon exome sequencing of the 160 genes implicated in cancer using the Illumina MiSeq sequencing platform (Illumina, San Diego, CA). The 160 genes examined are listed in Appendix Table A2. The smallest quantity of DNA had a DIN > 3.1. Sequencing data were entered into the GenomeJack bioinformatics pipeline (Mitsubishi Space Software, Tokyo, Japan) for analysis. Cancer-specific changes in somatic genes, including single-nucleotide variants, insertions/deletions, and copy-number variations were detected and used to determine the tumor mutation burden. In our system, secondary germ line findings could be identified by comparing the genomic profiles obtained for tumor tissues and peripheral blood mononuclear cells.
